# NETest and Gastro-Entero-Pancreatic Neuroendocrine Tumors: Still Far from Routine Clinical Application? A Systematic Review

**DOI:** 10.3390/genes16020161

**Published:** 2025-01-27

**Authors:** Roberta Elisa Rossi, Anna La Salvia

**Affiliations:** 1 Gastroenterology and Endoscopy Unit, IRCCS Humanitas Research Hospital, Via Manzoni 56, Rozzano, 20089 Milan, Italy; robertaelisa.rossi@gmail.com; 2 National Center for Drug Research and Evaluation, National Institute of Health Viale Regina Elena 299, 00161 Rome, Italy

**Keywords:** NETest, diagnosis, prognosis, gastro-entero-pancreatic neuroendocrine tumors, personalized medicine

## Abstract

**Background**: Gastro-entero-pancreatic neuroendocrine tumors (GEP-NETs) are the most prevalent subgroup among NETs and include heterogeneous tumors characterized by different clinical behavior and prognosis. The NETest is a tool based on real-time PCR combined with deep learning strategies to specifically identify tumors with a neuroendocrine genotype. Despite the promising results achieved regarding its utility in the field of GEP-NETs, the NETest has not yet entered into routine clinical practice. **Methods**: We performed a systematic review aimed at summarizing available evidence on the application of the NETest in both the diagnosis and the prognostic stratification of GEP-NETs. **Results**: We identified five studies evaluating the diagnostic role of the NETest and nine studies evaluating its prognostic value. The NETest emerged as a reliable biomarker for GEP-NET diagnosis with an accuracy higher than 90%, regardless of tumor stage and grade. However, according to some studies, the NETest showed a low specificity, mainly attributed to interferences with other gastro-intestinal malignancies. In terms of prognostic value, the NETest correlated with the detection of residual disease after surgery in six studies. The NETest was also associated with patients’ survival outcomes, namely progression-free survival (PFS) and overall survival (OS) in three studies. **Conclusions**: According to current systematic review, the value of the NETest both for diagnosis and for prognosis of GEP-NET emerged as robust across different studies. Further prospective analysis on larger GEP-NET series is encouraged to validate this tool, improving patients’ diagnosis, management, and follow-up.

## 1. Introduction

Neuroendocrine tumors (NETs) are malignant neoplasms originating from neuroendocrine cells which can occur throughout the whole body. Gastro-entero-pancreatic NETs (GEP-NETs) are the most prevalent subgroup (accounting for more than 60% of well-differentiated NETs) and include heterogeneous tumors characterized by different clinical behavior and prognosis. The incidence of GEP-NETs has significantly increased worldwide, likely due to better disease awareness and improvements in diagnostic tools [1].

The diagnostic pathway of GEP-NETs is still challenging due to their heterogeneity, morphogenic and clinical features, as well as the absence of widely available circulating biomarkers.

According to the 2022 WHO classification, GEP-NETs are classified as grade 1 (G1), grade 2 (G2), and grade 3 (G3) tumors based on a progressively increasing proliferation index [2]. The biological and clinical aggressiveness of these tumors increases with the tumor grade. However, the clinical presentation and the disease course are highly variable beyond the histopathological grade.

Furthermore, GEP-NETs are highly heterogeneous tumors also in terms of prognosis. Few key prognostic factors have been identified, such as age, sex, tumor stage (according to the TNM system), tumor grade, and tumor primary origin. Regarding the last factor, NETs in the rectum (24.6 years) and appendix (>30.0 years) had the best median overall survival (OS) among site groups, while NETs in the pancreas (3.6 years) have been found to have the worst median OS [1].

The precise mechanisms underlying the occurrence of these tumors are still far from being clearly understood. Of note, a family history of any cancer, smoking, and previous cholecystectomy have been suggested as potentialrisk factors. An Italian multicenter study reported that family history of colorectal and breast cancer as well as smoking appear to be risk factors for the development of small bowel NETs, while the use of aspirin might be a protective factor [3]. Regarding the pancreatic counterpart, a family history of pancreatic adenocarcinoma, chronic pancreatitis, high alcohol intake, and recent-onset diabetes have all been reported as possible risk factors [4]. It is, therefore, likely that mechanisms of carcinogenesis for endocrine cells in different sites can be specific and similar to those of their exocrine counterparts; however, available data are still insufficient to draw robust conclusions.

In the precision medicine era, the use of a combination of biomarkers and statistical models to integrate different variables to improve patients’ stratification has become progressively more common. In this context, nomograms are reliable tools, consisting of intuitive graphs of statistical predictive models that are able to quantify the risk of a clinical event (e.g., they are able to stratify those patients affected by a specific cancer type according to survival outcomes). Several nomograms have been developed to predict treatment efficacy or OS, but a measure of the underlying tumor biology that reflects tumor development is still not available [5,6,7,8,9], which affects the ability to provide a tailored management to each single GEP-NET patient, according to both the patient’s and tumor’s features.

With regard to tumor biomarkers, unfortunately, to date any single circulating biomarker has been identified as clinically significant and useful in the diagnosis in the detection of patients presenting a residual disease after surgery performed with curative intent and in prognosis stratification of patients.

The multi-analyte circulating biomarkers demonstrate promising advantages in the field. In this context, the NETest is a multi-analyte algorithmic biomarker based on a real-time polymerase chain reaction (PCR) combined with deep learning strategies to specifically identify tumors with a neuroendocrine genotype [10,11,12]. In detail, the test uses a two-step protocol (mRNA isolation, cDNA production, and PCR). mRNA is isolated from ethylenediaminetetraacetic acid (EDTA)-collected whole blood samples, and real-time PCR is performed to interrogate 51 genes with the aid of four different prediction algorithms [13]. Blood gene expression of the 51 markers is normalized to housekeepers and quantified versus a population control. NETest results correspond to a range between 0 and 100%, being these values directly proportional to disease activity levels at the time of testing. The scores of 0–40% defines low activity and is associated with tumor stability, whereas the levels of 41–79% and ≥80% correlate with moderate or high activity and with tumor progression. It is, therefore, clear that the NETest is a valid tool in the diagnostic phase, but, even more important, it plays a useful role as a prognostic marker [13].

Current review is aimed at systematically summarizing available evidence on the application of the NETest in both the diagnosis and the prognostic stratification of GEP-NETs to better understand whether it is a handy tool in everyday clinical practice or if its applications remain limited to specific settings and scientific purposes only.

## 2. Materials and Methods

This systematic review was performed in accordance with the Preferred Reporting Items for Systematic Reviews and Meta-analysis (PRISMA) recommendations [14]. A literature search was performed in PubMed, with the search strategy last updated in December 2024. The following terms were used for the literature search: gastro-entero-pancreatic neuroendocrine tumors; gastro-entero-pancreatic neuroendocrine neoplasms; NETest; diagnosis; prognosis.

All the available studies, published between 2010 and December 2024 in English, were considered. Studies that were deemed as potentially eligible were retrieved and evaluated as full text. The excluded studies together withthe reasons for exclusion were recorded. When available, key data were extrapolated from each study: total number of patients, study design (i.e., prospective vs. retrospective), diagnostic accuracy of NETest, the prognostic role of NETest in GEP-NETs.

## 3. Results

**Figure 1:**
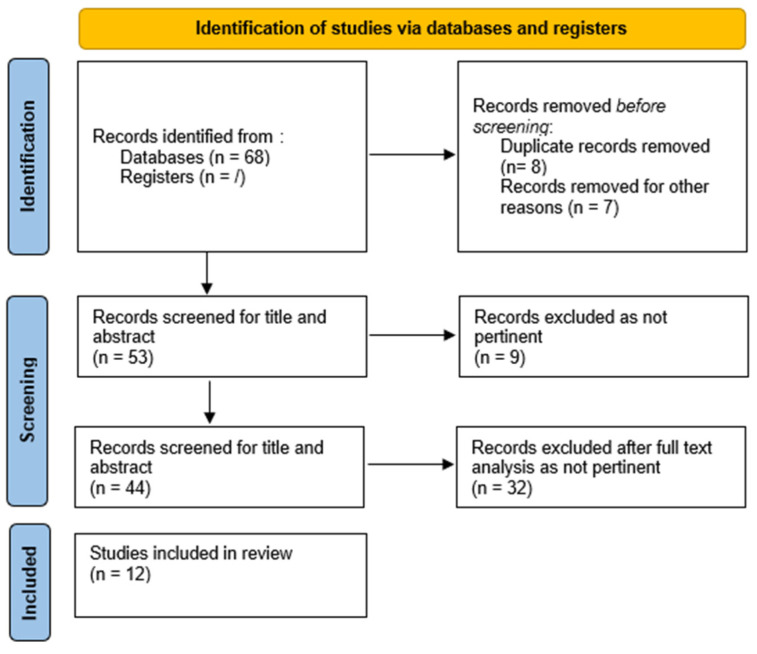
Study selection flowchart according to PRISMA recommendations.

With our literature search we found 68 studies using the following keywords “neuroendocrine tumors” or “neuroendocrine neoplasms” or “carcinoids” AND NETest. After removing duplicates, 12 studies met the inclusion criteria for the purpose of our systematic review and were analyzed as full texts. The other studies were discarded due to insufficient or not pertinent data. The study selection flowchart is provided in Figure 1.

### 3.1. Diagnostic Role of NETest in Gastro-Entero-Pancreatic Neuroendocrine Tumors

The NETest has progressively gained increasing popularity as a useful tool for detecting the presence of NETs of different origins, as small bowel and pancreatic primaries, with an accuracy higher than 90% regardless of tumor stage and grade [15]. According to a recent multicenter study including three different subgroups of NETs (not only GEP-NETs, but also lung NETs, and of unknown origin), the NETest was able to discriminate NETs from a complex set of controls: healthy, non-NET malignancies, and benign diseases responsible for increased Chromogranin A (CgA) levels [16]. Of note, the NETest was found to perform better compared to CgA (>91% vs. <50%, respectively). This study had several strengths, being a multicenter, multinational experience of the use of NETest in clinical practice in ENETs Centers of Excellence. Moreover, all samples were deidentified and analyzed blind by a central USA CLIA-approved laboratory. On the other hand, study limitations include heterogeneity of imaging studies, the absence of centralized pathological review, and dependence on individual centers to provide clinical information.

A recent meta-analysis of ten different studies [17] reported the diagnostic accuracy of the NETest to be 95–96%, highlighting its important role as a diagnostic tool. Conversely, according to a large independent validation study [18], including 140 consecutive GEP-NET patients and 113 healthy volunteers, the median NETest score levels in NET patients was 33 compared to 13% in controls. Of note, sensitivity and specificity accounted for 93 and 56%, respectively, compared to a sensitivity and specificity of CgA of 56 and 83%, respectively. Based on these findings, the authors concluded that the low specificity of the NETest precludes its use as a screening test for GEP-NETs. A possible explanation of this disappointing specificity may be due to both a possible interference of gene expression caused by non-malignant conditions and the presence of platelets and extracellular RNA in the source of the transcripts [18]. Similar results have also been reported by Al-Toubah et al. [19] in their study including 49 patients with metastatic NETs, 21 patients with other metastatic gastro-intestinal cancers, and 26 healthy individuals. Using 13% as the upper limit of normal, the sensitivity of the NETest was 98% with an overall specificity of 66% (95% CI 51–79%), with 16 false-positive results. Again, the low specificity was mainly attributed to interferences with other gastro-intestinal malignancies. The main study limits can be identified as its relatively small sample size and the collection of single blood sample. On the contrary, the evaluation of the NETest score in non-NET malignancies patients could be evaluated as potentially useful to define the role of NETest in these tumors.

A recent prospective study [20] focused on high-grade (HG) GEP-NETs. The NETest was performed prior to the start of chemotherapy in 60 patients who were histologically re-classified as follows: 45 cases were classified as HG GEP-NET (12 cases as NET G3, 30 as neuroendocrine carcinoma-NEC, and 3 cases with ambiguous morphology); 1 case was re-classified as a small intestinal NET G2 with Ki-67 of 18%; 8 cases were classified as mixed non-neuroendocrine and neuroendocrine neoplasm (MiNEN), 3 cases as adenocarcinomas with neuroendocrine differentiation (ADNE) with intense synaptophysin staining, and an additional 3 cases re-classified as adenocarcinomas. The NETest was almost always elevated in NECs, and inNET G3 and MiNEN, as well. Unfortunately, the small sample size prevents to draw robust conclusions. Of note, another relevant limitation was the evaluation only of abaseline blood sample.. It is, however, important to point out that this is the first study to analyze the NETest in the specific subgroup of HG HEP-NETs; furthermore, other strengths of the study were the collection of blood samples before the start of any treatment, and the pathologic review and re-classification in line with the WHO 2019 classification.

In a Japanese study [21], among 35 GEP-NET patients, the diagnostic sensitivity of the NETest was 97.1%, showing high diagnostic efficacy. As a point of strength, this is the first study analyzing the NETest in a Japanese population; however, some limitations are present, including sample damage or delays, the relatively small number of cases, and the short observation period. As a future perspective, it is necessary to establish local laboratories to run these assays to make the results more accurate and also efficient in other populations.

### 3.2. Prognostic Role of NETest in Gastro-Entero-Pancreatic Neuroendocrine Tumors

In the field of GEP-NETs, the identification of innovative and reliable prognostic tools represents an unmet clinical need, considering that traditional biomarkers such as CgA have failed to be recognized as clinically useful markers [22]. Within this context, it has been shown that the post-operative recurrence in NET patients could be predicted with the NETest [16]. Different NETest results were observed after palliative R1/R2 surgery (n = 51), where in 100% of the cases, NETest levels remained elevated, versus curative R0 surgery (n = 102), where NETest levels were normal in 81 patients (70%) with no recurrence at 2 years. In the 31 patients (30%) with elevated levels after R0 surgery, 25 (81%) recurred within 2 years. In addition, the NETest was able to detect progressive disease (95%) when compared to CgA (57%, *p* < 0.0001). The strengths of this study were the large sample size (1684 NET cases) together with the study design, which was an observational, prospective, cross-sectional, multicenter, multinational and comparative cohort study. The main limits of this study were the heterogeneity of imaging reports, the lack of a centralized pathological review, and the non-centralized collection and revision of clinical data of included patients. Another study confirmed these results, showing that surgical resection of the tumor significantly decreased NETest scores, while an elevated NETest predicted radiological disease recurrence with 94% accuracy [23]. In this study an evaluation of over 24 months after tumor resections was carried out. In addition, a robust statistical analysis was performed. A limitation of this study was the heterogeneity of NET patients (with 47 pancreatic, 26 small intestinal, 26 lung, 2 appendiceal, 1 duodenal, 1 gastric primary origins). In the same direction, another prospective study including 13 small bowel NETs demonstrated that NETest scores significantly decreased after curative surgery (*p* < 0.05) [24]. In the group of patients with progressive disease, a high NETest score was present in three cases (60%) and an intermediate NETest score in the remainder (40%). A prospective study including 21 lung NETs showed a normalization of NETest levels after surgical removal in cases with R0 resection (*p* < 0.001) [25]. Moreover, the NETest differentiated progressive disease (73%) from stable disease (36%, *p* < 0.001), suggesting a role of the NETest also in lung NETs, in line with GEP-NETs. The main limit of both of these studies [24,25] was the small sample size, while the prospective design was an added value.

A study by Partelli and colleagues, including 30 NETs of pancreatic origin treated with surgery, demonstrated that a decrease in NETest levels after resection was associated with surgical efficacy, whereas CgA had no clinical utility [26]. The NETest positively correlated with recurrence in a study of pancreatic NET patients undergoing surgery [27]. The NETest scores were higher in the 11 patients who underwent radical resection with recurrence (56%) compared with the 12 R1 cases with no recurrence (39%) and the 12 R0 with no recurrence (28%, *p* < 0.005). In addition, logistic regression modeling of clinic-pathologic features had an accuracy of 83%, while including the NETest, the accuracy was higher, at 91%. Of note, the combination of clinic-pathological characteristics and the NETest was twice as effective as clinical and pathological aspects (increase in R2 value from 43% to 80%). The main strength of these two studies [26,27] was the homogeneity of the study population, all of which were of pancreatic origin and underwent surgical resection of the primary tumor. Among the limits, this was a single-center study, and there were small sample sizes in both cases.

Another study showed that the NETest could predict radiological disease progression with a sensitivity of 42.9% or 40.9% and a specificity of 91.5% or 86.7%, using optimal cut-off values of 76.7% or 56.7%, respectively [28]. In the same study, NETest scores were associated with progression-free survival (PFS) and OS in patients with metastatic small intestinal NETs [28]. A major limitation of this study was that only 16 cases were post-operatively disease-free according to 68Ga-DOTATATE PET versus 102 metastatic small intestinal NETs, whereas the strengths were that all patients had the same primary tumor origin and the robustness of the statistical plan applied. The data coming from the registry of NET patients collected for the study, RegisterNET (NCT02270567) demonstrated that the NETest was the only variable associated with outcome (multiple regression: coefficient, 0.32; *p* < 0.001; logistic regression: odds ratio [OR], 6.1; *p* < 0.001) [29]. Analysis using Cox proportional modeling confirmed that the NETest was the only variable related to outcome (*p* < 0.0001). A total of 100 NET patients with pathological confirmation were enrolled over a period of 22 months, with 45% of cases under observation and 55% in a treatment cohort. One strength of this study was that each patient group was analyzed separately. Limitations related to registry studies, however, may include the quality of the data and how survival outcomes are measured. Interestingly, Sorbye and colleagues evaluated the role of the NET test in a population of advanced HG GEP-NENs [20]. In this observational study, survival outcomes of the evaluable patients treated with chemotherapy (n = 39) were significantly reduced in cases with the NETest > 60 (OS 6.5 months vs. 10.2 months and PFS 4.1 months vs. 5.6 months, respectively). This study also suggests a role in the field of HG GEP-NENs, as demonstrated in well-differentiated tumors. However, the study population is highly heterogeneous (including poorly differentiated carcinomas but also G3 NET, as well as tumors with different primary origins), and this is a limit together with an overall small sample size.

The main characteristics of the studies included in our systematic review are summarized in Table 1.

## 4. Discussion

The NETest is a promising tool in the field of GEP-NETs, providing a noninvasive, innovative, and reliable biomarker to improve the diagnosis, management, and prognosis stratification of GEP-NETs [30,31]. To date, several clinical experiences, both prospective and retrospective, have demonstrated its utility, but further data on larger populations of GEP-NETs are probably needed to validate this biomarker and lead to its routine application in the real-world scenario, also considering its high costs.

The diagnostic pathway of GEP-NETs is still challenging due to their heterogeneity, morphogenic and clinical features, as well as the absence of widely available circulating biomarkers; thus, the identification of a reliable diagnostic and prognostic tool is of utmost importance.

According to available studies, the NETest has progressively emerged as a reliable diagnostic tool with an accuracy rate > 90% regardless of the stage or grade of the tumor [16]. The possibility of having a diagnostic effective screening and a way to discriminate GEP-NET patients from healthy controls is crucial. However, there are conflicting results as, according to some authors [16,19], the NETest showed a low specificity, mainly attributed to interferences with other gastro-intestinal malignancies, which might limit its routine clinical use in the diagnostic setting. However, most available studies highlight its accuracy in the diagnostic setting and its superiority to available biomarkers (i.e., CgA). As a future perspective, further studies are warranted to better define the actual role of the NETest in the diagnosis of GEP-NETs.

Overall, the included studies assessing the prognostic value of the NETest showed the value of this tool in detecting residual disease after surgery across different primary origins of the tumors (such as small bowel, pancreas, and lung) [16,23,24,25,26,27]. The NETest demonstrated to correlate with radical versus palliative surgery. This issue could have relevant implications for clinical practice, considering that surgery still remains the unique curative therapeutic strategy for NETs and, therefore, the possibility of the early identification of residual disease after surgery (by using a noninvasive tool such as a blood test) would have a crucial role in a prompt intervention.

In addition, it has been suggested that monitoring NET transcript levels in blood can identify progressive disease, thus potentially representing an essential addition in the multidisciplinary decision-making strategy [28]. The NETest is a quantitative test that measures specific biomarkers associated with NETs, providing critical insights into the tumor’s biology. From a pathophysiological standpoint, higher NETest scores reflect increased tumor burden, with a direct correlation between elevated biomarker levels and both tumor volume and metastatic spread. As the scores increase, it indicates a more aggressive tumor phenotype characterized by enhanced proliferation, angiogenesis, and the ability to invade surrounding tissues. This heightened biological activity results in larger tumor sizes and a greater likelihood of metastasis to distant organs, such as the liver and lymph nodes. Consequently, higher NETest scores are strongly predictive of poor prognosis, as they signify not only an advanced disease stage but also a higher potential for rapid progression [16].

The evaluation of progressive disease (PD), indeed, according to international guidelines, has been traditionally assessed by evaluating morphologic criteria at diagnostic imaging. In this setting, the validated Response Evaluation Criteria in Solid Tumors (RECIST) version 1.1 still represents the standard of care in oncology, including the neuroendocrine setting [32]. Therefore, the disease course and response to treatment (including PD) is strictly dependent on imaging techniques, so far. The NETest could represent a noninvasive and quick method to obtain a prompt monitoring of treatment outcomes, eventually anticipating and weighing the benefits of treatments, especially in terms of survival gain. In addition, considering the peculiar features of NETs (e.g., the overall low proliferation index and high vascularization), the assessment of response through the morphologic criteria according to RECIST v 1.1 has been questioned. An interesting retrospective multicentric study explored alternative criteria using different numbers of measured liver metastases from NETs and thresholds of size and density variation [33]. Thus, even in this light, the NETest could be a way to overcome these limitations, as a potentially standardized and homogeneous measure in all NET patients (independently from tumor vascularization or grade).

Few studies [20,28,29] have also detected a statistically significant association between NETest results and patients’ survival outcomes, namely PFS and OS. This association is clinically significant and potentially relevant for daily clinical practice, suggesting a personalized way to stratify patients, and thus, improve their management. In addition, the opportunity to have a repeatable method to follow the clinical course of a GEP-NET patient may be useful in the decision-making process, together with a response to treatment assessment by morphological and functional imaging, patients’ symptoms, quality of life, and preferences.

Limitations of current systematic review include the small sample size of the majority of the included studies, their heterogeneity, and their somewhat conflicting results; however, we performed a systematic review following the PRISMA recommendations, trying to provide clear information regarding the application of the NETest in the neuroendocrine setting, also highlighting its limitations.

## 5. Conclusions

According to this systematic review, the value of the NETest both for diagnosis and for prognosis of GEP-NETs emerged as robust across different studies. Further prospective analysis on a larger GEP-NET series is encouraged to validate this tool, improving patients’ diagnosis, management, and follow-up.

## 6. Take Home Messages

1. The NETest appears to be a useful tool in the diagnosis of NETs of different origins, including the pancreas, small intestine, and even those of unknown origin, with an accuracy of more than 90% regardless of the stage or grade of the tumor.
2. The NETest also seems to be a relevant prognostic tool.
3. First, the NETest showed a value in detecting residual disease after surgery, across different primary origins of the tumors (including the small bowel and pancreas)
4. In addition, the NETest has been suggested to be able to identify progressive disease; thus, it could represent a noninvasive and quick method to obtain a prompt monitoring of treatment outcomes.
5. Few studies have also detected a statistically significant association between NETest results and patients’ survival outcomes, including PFS and OS.
6. All considered, the NETest could represent a relevant tool for daily clinical practice, suggesting a personalized way to stratify and manage GEP-NET patients.
7. Taking into account the intrinsic limitations of available studies and the NETest-related costs, further studies are warranted to better delineate the actual role of the NETest in the management of GEP-NETs and to promote its application in everyday clinical practice.

## Figures and Tables

**Table 1:** Studies evaluating a diagnostic or prognostic role of NETest in GEP-NET.

Author, Year	Type of Study	Diagnostic or Prognostic Role of NETest	Number of NET Patients Included
Modlin IM, 2021 [16]	Prospective	Diagnostic, Prognostic	1684
van Treijen MJC, 2018 [18]	Prospective	Diagnostic	140
Al-Toubah T, 2021 [19]	Prospective	Diagnostic	49
Sorbye H, 2024 [20]	Prospective	Diagnostic, Prognostic	60
Zhang H, 2024 [21]	Prospective	Diagnostic	35
Modlin IM, 2021 [23]	Prospective	Prognostic	103
Laskaratos FM, 2020 [24]	Prospective	Prognostic	13
Filosso PL, 2018 [25]	Prospective	Prognostic	21
Partelli S, 2020 [26]	Prospective	Prognostic	30
Genç CG, 2018 [27]	Prospective	Prognostic	35
Gertner J, 2024 [28]	Prospective	Prognostic	118
Liu E, 2019 [29]	Prospective	Prognostic	100
